# Improved prediction of gestational hypertension by inclusion of placental growth factor and pregnancy associated plasma protein-a in a sample of Ghanaian women

**DOI:** 10.1186/s12978-018-0492-9

**Published:** 2018-03-27

**Authors:** Edward Antwi, Kerstin Klipstein-Grobusch, Joyce L. Browne, Peter C. Schielen, Kwadwo A. Koram, Irene A. Agyepong, Diederick E. Grobbee

**Affiliations:** 1 Julius Global Health, Julius Center for Health Sciences and Primary Care, University Medical Center Utrecht, Utrecht University, Utrecht, the Netherlands; 2 Ghana Health Service, P.M.B, Ministries, Accra, Greater Accra Ghana; 3 Noguchi Memorial Institute for Medical Research, College of Health Sciences, University of Ghana, Legon, Accra Ghana; 4 Division of Epidemiology & Biostatistics, School of Public Health, Faculty of Health Sciences, University of the Witwatersrand, Johannesburg, South Africa; 5 Center for Infectious Diseases Research, Diagnostics and Screening (IDS), National Institute for Public Health and the Environment (RIVM), Bilthoven, the Netherlands

**Keywords:** Prediction model, Gestational hypertension, Biomarkers, Hypertensive disorders of pregnancy

## Abstract

**Background:**

We assessed whether adding the biomarkers Pregnancy Associated Plasma Protein-A (PAPP-A) and Placental Growth Factor (PlGF) to maternal clinical characteristics improved the prediction of a previously developed model for gestational hypertension in a cohort of Ghanaian pregnant women.

**Methods:**

This study was nested in a prospective cohort of 1010 pregnant women attending antenatal clinics in two public hospitals in Accra, Ghana. Pregnant women who were normotensive, at a gestational age at recruitment of between 8 and 13 weeks and provided a blood sample for biomarker analysis were eligible for inclusion. From serum, biomarkers PAPP-A and PlGF concentrations were measured by the AutoDELFIA immunoassay method and multiple of the median (MoM) values corrected for gestational age (PAPP-A and PlGF) and maternal weight (PAPP-A) were calculated. To obtain prediction models, these biomarkers were included with clinical predictors maternal weight, height, diastolic blood pressure, a previous history of gestational hypertension, history of hypertension in parents and parity in a logistic regression to obtain prediction models. The Area Under the Receiver Operating Characteristic Curve (AUC) was used to assess the predictive ability of the models.

**Results:**

Three hundred and seventy three women participated in this study. The area under the curve (AUC) of the model with only maternal clinical characteristics was 0.75 (0.64–0.86) and 0.89(0.73–1.00) for multiparous and primigravid women respectively. The AUCs after inclusion of both PAPP-A and PlGF were 0.82 (0.74–0.89) and 0.95 (0.87–1.00) for multiparous and primigravid women respectively.

**Conclusion:**

Adding the biomarkers PAPP-A and PlGF to maternal characteristics to a prediction model for gestational hypertension in a cohort of Ghanaian pregnant women improved predictive ability. Further research using larger sample sizes in similar settings to validate these findings is recommended.

## Plain English summary

Gestational hypertension and preeclampsia affect between 5 to 10% of all pregnancies and can result in complications in the mother and the fetus. Early prediction of pregnant women at risk of these conditions will lead to better monitoring and appropriate management. This study was conducted in antenatal clinic settings in Ghana to investigate whether adding two biomarkers, placental growth factor and pregnancy associated plasma protein A, to a previously developed prediction model based on maternal clinical characteristics improved the performance of the model.

Logistic regression was used to derive a prediction model. Adding biomarkers to a previously validated prediction model improved the performance of the model for gestational hypertension.

We recommend further research using larger sample sizes in similar settings to validate our findings.

## Background

Hypertensive disorders of pregnancy (HDP) are leading causes of maternal morbidity and mortality globally and affect about 5 to 10% of all pregnancies [1, 2]. The burden of these conditions is greatest in low and middle income countries (LMICs) [3, 4]. Early identification of pregnant women at risk of developing these conditions result in better monitoring and management to minimize complications to the mother and the fetus. Prediction models have been used to identify women at high risk of HDPs, particularly preeclampsia [3–6]. In addition, prevention interventions could be started such as calcium and aspirin supplementation that have been shown to reduce the risk of HDPs, particularly preeclampsia [7–12]. For example, in the ASPRE (Combined Multimarker Screening and Randomized Patient Treatment with Aspirin for Evidence-Based Preeclampsia Prevention) trial with risk selection based on screening, a reduction in the incidence of preterm preeclampsia in the aspirin arm by 62% was observed [12].

PAPP-A is a protease that is involved in the local release of insulin-like growth factors. Low first trimester levels of PAPP-A is associated with hypertensive disorders of pregnancy [13–15]. Placental growth factor (PIGF) is an angiogenic factor and low concentrations have been observed in pregnant women who develop preeclampsia. Suboptimal secretion of PlGF between 8 to 14 weeks gestation as a result of placental dysfunction has been associated with disorders such as preeclampsia, intrauterine growth restriction, small-for-gestational age and still births [16].

The aim of this study was to assess whether the addition of the biomarkers, placental growth factor (PIGF) and pregnancy-associated protein A (PAPP-A) to a previously developed prediction model [17] based on maternal clinical characteristics (diastolic blood pressure, family history of hypertension in parents, history of gestational hypertension (GH) in a previous pregnancy, parity, height and weight) improved prediction of gestational hypertension.

## Methods

### Study design and study population

This study was nested in a prospective cohort of 1010 adult pregnant women with a singleton pregnancy and without known pre-existent hypertension recruited between July 2012 and March 2014 at Ridge Regional Hospital and Maamobi General Hospital in Accra. Accra, the capital city of Ghana, is cosmopolitan with high, middle and low-income persons from different ethnic backgrounds living and working in the city [18]. Persons from all the social strata access health services, including antenatal and delivery care in these public hospitals. These hospitals were also chosen because they have a high attendance so the recruitment of pregnant women into the study could be completed in a shorter time. Eligibility criteria for this study were gestational age at enrollment of between between 8 and 13 weeks, based on ultrasound scan. This specific subset of women was selected based on evidence that prediction with these biomarkers is most appropriate at this gestational age [7–10, 19–21]. Women with gestational age at enrollment of less than 8 weeks or more than 13 weeks (*n* = 411), without PlGF MoM values (*n* = 95) or women without outcome data (*n* = 131) were excluded. We used the principle of 10 outcome events per variable for logistic and Cox regression analysis [22–25] to obtain a sample size adequate for our analysis. With an incidence of gestational hypertension of 10% in the Ghanaian population [26], and eight variables in the prediction model, a sample size of 393 women was considered adequate for the analysis.

The women were included in the study after they had given written informed consent and were interviewed by trained research assistants using a structured questionnaire for socio-demographic characteristics and obstetric history. They were followed up at each antenatal clinic visit till they delivered. None of the women who developed gestational hypertension progressed to preeclampsia. Pregnancy outcomes were obtained at delivery and from the hospital maternity register.

## Variables

### Independent variables

Maternal weight (measured in kilogrammes with a bathroom scale), height (measured in centimeters with a stadiometer), blood pressure (measured in millimeters of mercury) and urine protein (defined as 2+ or more on urine dipstick) were obtained at the initial and subsequent antenatal clinic visits from the maternal health record books.

Blood pressure measurements were performed by trained midwives using a mercury sphygmomanometer. The appropriate adult sized cuff was placed on the bare left upper arm with the woman comfortably seated and her back supported and legs uncrossed. The arm was at the level of the heart and neither the patient nor the observer talked during the measurement. Korotkoff phase V sounds were used [27]. Two readings were taken at interval of five minutes and the average used as the woman’s blood pressure.

### PAPP-A and PlGF assay

Blood specimen was obtained from women on the day of their enrollment into the study by a phlebotomist. After the blood had coagulated, it was centrifuged to obtain the serum which was stored at a temperature of -20 °C in a freezer at the Maamobi General Hospital. Serum samples from the Ridge Hospital were stored temporarily in a fridge at 4 °C and transported daily in a cold box with ice packs to the laboratory at Maamobi General Hospital for storage. The frozen serum samples were air-freighted on dried ice to the Dutch Institute for Public Health and Environment (RIVM) in Bilthoven, the Netherlands, where they were stored at a temperature of − 80 °C until they were analyzed for PIGF and PAPP-A. PAPP-A and PlGF concentrations were determined using commercially available immunoassays and the AutoDelfia automated analyzer (PerkinElmer, Turku, Finland). Details of the assay method are described elsewhere by Browne et al. [28]. PAPP-A concentrations were corrected for gestational age and maternal weight and expressed as multiple of the median (MoM) using the reference equations from the Dutch national prenatal screening programme for Down syndrome based on PAPP-A measurements between 8 to 13 weeks gestation of more than 10,000 pregnancies [29]:

PAPP-A MoM gestational age correction

$$ y=12,605.9606\hbox{--} {552.53697}^{\ast}\times +{7.42649}^{\ast }{\times}^2\hbox{--} {0.0278}^{\ast }{\times}^3, $$

where x = gestational age at blood sampling in days.

PAPP-A MoM weight correction; Exp (1.23234075–0.0181912*x), where x = weight in kilograms.

PlGF concentrations were also corrected for gestational age and expressed as MoM [28] by using the manufacturer’s (Perkin Elmer) reference equation for gestational age in days (between 9 to 13 weeks gestation) as follows:

$$ y=75.08\hbox{--} {1.7769}^{\ast}\times +{0.01589}^{\ast }{\times}^2 $$

where x = gestational age at blood sampling in days.

PlGF was not corrected for maternal weight because serum PlGF concentration is not correlated with maternal weight [29].

### Outcome

The outcome, gestational hypertension, was defined as a systolic BP of 140 mmHg or more and or a diastolic BP of 90 mmHg or more on at least two separate occasions, and present for the first time after 20 weeks of pregnancy [30].

### Ethical considerations

Ethical approval for the study was granted by the Ethical Review Committee of the Ghana Health Service (GHS-ERC 07/09/11). All participating women gave written informed consent before they were enrolled in the study.

### Statistical analysis

SPSS software (version 20.0, IBM SPSS Statistics Inc., Chicago, Illinois, USA) and R statistical software (R version 3.1.0 (2014–04-10). The R Foundation for Statistical Computing Platform: x86_64-w64-mingw32/× 64 (64-bit)) were used for statistical analysis. The mean and standard deviation of continuous predictors were calculated for women who developed gestational hypertension and those who did not. Means were compared using the Student’s t-test; percentages for categorical data were assessed by Chi-square test. The median with interquartile range was reported for non-normally distributed variables.

Logistic regression was used to derive the original prediction model using gestational hypertension as the outcome and the following maternal clinical characteristics as the predictors: maternal height, weight, parity, previous history of gestational hypertension, family history of hypertension and diastolic blood pressure. The maternal weight, height, diastolic blood pressure, parity, PAPP-A MoM and PlGF MoM were included in the logistic regression model as continuous variables. The principle of 10 events per variable for logistic and Cox regression analysis [31] was applied in model building. A history of hypertension in parents and history of gestational hypertension in a previous pregnancy were included in the logistic regression as dichotomous variables. As the variable ‘previous history of gestational hypertension’ was not applicable to primigravid women, a separate model was fitted for them.

PAPP-A MoM and PlGF MoM were included in the model as continuous variables so as not to lose power through categorization, and also because the appropriate cut-off value of these biomarkers for the Ghanaian population is not known [28]. The PAPP-A and PlGF as MoM values were included in turns and then together to the logistic regression. The predictive ability of each model (PAPP-A only, PlGF only, combined) was assessed. The models were internally validated using the bootstrapping technique. The resulting shrinkage factor after bootstrapping was used to adjust the regression coefficients, thus correcting for model overfitting.

The performance of the models was assessed by the area under the receiver operating characteristic curve (AUC) or c-statistic. The AUC of the original model with only maternal clinical characteristics was compared to that of the models with PAPPA and maternal clinical characteristics, PlGF with maternal characteristics and both PAPP-A, PlGF and maternal characteristics.

## Results

Characteristics of the 373 study participants are presented in Table 1. Most of the women (81%) were multiparous. The mean age was 28.3 (SD 4.9) years and the mean gestational age at booking was 11.6 weeks (SD 1.4).

**Table 1:** Baseline characteristics of the study population (*n* = 373)

Variable	Mean (SD) or N (%)
Age (years)	28.3 (4.9)
Height (cm)	161.2 (6.3)
Weight (kg)	66.5 (13.3)
Systolic blood pressure (mmHg)	110.5 (12.9)
Diastolic blood pressure (mmHg)	68.9 (10.3)
Gestational age at booking (weeks)	11.6 (1.4)
PlGF MoM corrected for gestational age	Median 1.28, IQR (0.96–1.88)
PAPP-A MoM corrected for gestational age	Median 2.29, IQR (1.15–3.86)
PAPP-A MoM corrected for gestational age and maternal weight	Median 2.34, IQR (1.19–3.82)
Parity:	
Primigravid women	71 (19.0%)
2–3 pregnancies	116 (31.1%)
> 4 pregnancies	186 (49.9%)

**Fig. 1:**
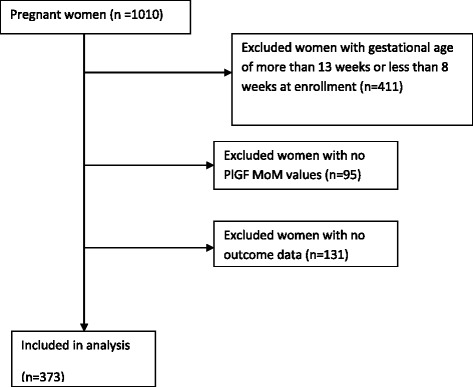
Flow chart illustrating participant selection

The flow chart for selection of study participants is shown in Fig. 1. Of 1010 women in the original cohort, 373 women met the inclusion criteria.

Table 2 compares characteristics of women who developed gestational hypertension to those who did not. Twenty-five women (6.7%) developed gestational hypertension. There was a difference in mean age between women who developed gestational hypertension and those who did not (30.3 (SD 5.3) years vs. 28.2 (SD 4.9) years, *p* = 0.04). There was no difference in mean height between women with and without gestational hypertension (159.1 cm (SD 7.1) vs. 161.4 cm (SD 6.3), *p* = 0.08). However, there was a difference in the mean weight of women with and without gestational hypertension (72.9 kg (SD 16.3) vs. 66.0 kg (SD 12.9), *p* = 0.013). The mean diastolic blood pressure differed between women who developed gestational hypertension and those who did not (74.3 mmHg (SD 13.6) vs. 68.5 mmHg (SD 9.9), *p* = 0.006).

**Table 2:** Baseline characteristics of the study population by the outcome, gestational hypertension

Variable (Mean (SD))	Gestational hypertension (No)	Gestational hypertension (Yes)	*p*-value
	*N* = 348	*N* = 25	
Age (years)	28.2 (4.9)	30.3 (5.3)	0.04
Height(cm)	161.4 (6.3)	159.1 (7.1)	0.08
Weight (kg)	66.0 (12.9)	72.9 (16.3)	0.013
Systolic blood pressure (mmHg)	110.1 (12.7)	116.4 (14.2)	0.018
Diastolic blood pressure (mmHg)	68.5 (9.9)	74.3 (13.6)	0.006
Gestational age at booking (weeks)	11.6 (1.4)	11.3 (1.5)	0.38

Table 3 presents the median and interquartile range of MoM of PAPP-A and PlGF by gestational week. The median MoM PAPP-A (adjusted for gestational age and maternal weight) ranged between 1.68 and 4.36. The median MoM PlGF ranged between 0.90 and 1.68.

**Table 3:** Median and interquartile range of MoM of PAPP-A and PlGF by gestational week (*n* = 373)

Gestational week	Number of women (%)	MoM PAPP-A, median (IQR), adjusted for gestational age and maternal weight	MoM PAPP-A, median (IQR), adjusted for maternal weight	MoM PlGF, median (IQR), adjusted for gestational age
8	17 (4.5)	4.36 (1.06–8.47)	4.46 (1.19–6.42)	1.17 (0.85–1.51)
9	40 (10.7)	1.68 (1.04–4.64)	2.04 (0.86–4.25)	0.90 (0.73–1.36)
10	86 (23.1)	2.39 (1.45–3.83)	2.33 (1.44–4.12)	1.15 (0.97–1.66)
11	71 (19.3)	1.76 (0.85–3.05)	1.96 (0.88–3.01)	1.21 (0.95–1.49)
12	66 (17.6)	2.21 (1.06–3.65)	2.26 (1.20–3.34)	1.29 (1.03–1.91)
13	93 (24.8)	2.63 (1.49–4.51)	2.55 (1.57–4.05)	1.68 (1.34–2.94)
Total	373	2.29 (1.15–3.86)	2.34 (1.19–3.82)	1.28 (0.96–1.88)

*IQR* Interquartile range, *MoM* multiple of the median

The median MoM value of the reference population by default is 1. The gestational age and weight adjusted PAPP-A median MoM was 2.29. The weight adjusted PAPP-A median MoM was 2.34 and the median PlGF MoM was 1.28

**Fig. 2:**
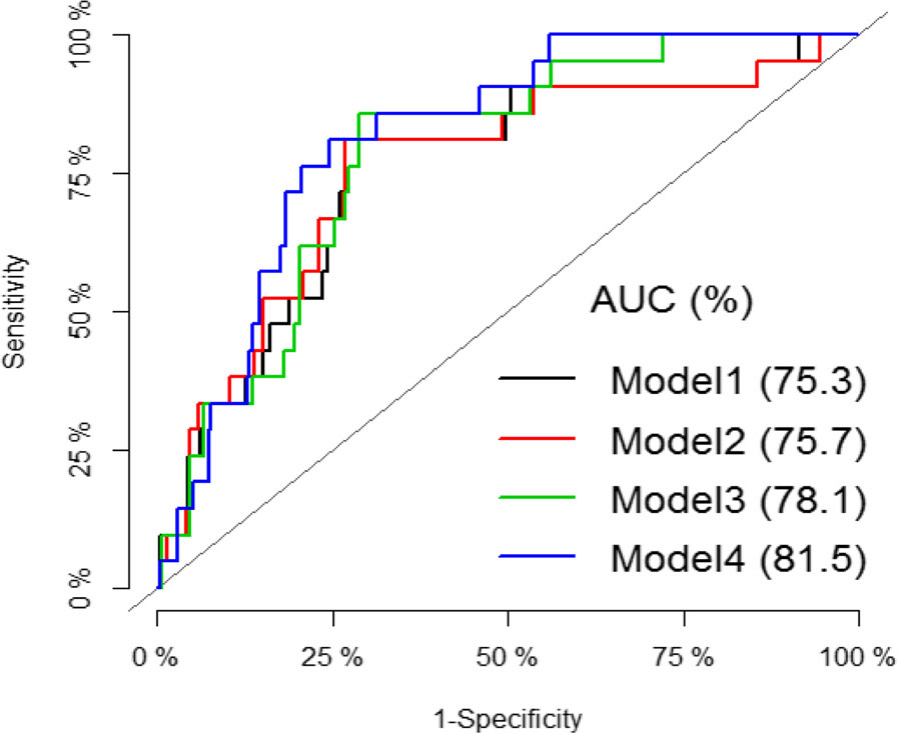
Receiver operating characteristic (ROC) curves of prediction models for multiparous women. Model 1 (black line): Maternal characteristics only, Model 2 (red line): Maternal characteristics and PlGF MoM, Model 2 (red line): Maternal characteristics and PlGF MoM, Model 2 (red line): Maternal characteristics and PlGF MoM

Table 4 shows the regression coefficients and the AUC of the various models for multiparous women. The AUC of the model with only maternal characteristics was 0.75 (0.64–0.86). The AUC of the model with maternal characteristics and PAPP-A was 0.78 (0.70–0.87), with maternal characteristics, and PlGF was 0.76 (0.64–0.87), and maternal characteristics with both biomarkers 0.82 (0.74–0.89). Figure 2 shows the Receiver Operating Characteristic curves for the prediction models for multiparous women. Table 5 shows the regression coefficients and the AUC of the models for primigravid women. The AUC of the model with only maternal characteristics was 0.89 (0.73- 1.00). The AUC of the model with maternal characteristics and both biomarkers was 0.95 (0.87-1.00).

**Table 4:** Regression coefficients and AUC of prediction models for multiparous women (*n* = 302)

Variable	Model with only maternal characteristics	Model with addition of PlGF MoM	Model with addition of PAPP-A MoM	Model with addition of PlGF MoM and PAPP-A MoM
Intercept	9.68	10.0	10.46	12.18
History of hypertension in parents	−1.52	−1.50	− 1.60	−1.65
Previous history of hypertension in pregnancy	0.47	0.55	0.42	0.72
Weight	0.026	0.025	0.024	0.023
Height	−0.097	−0.099	−0.102	− 0.112
Parity	0.29	0.29	0.33	0.34
Diastolic BP	0.036	0.036	0.037	0.042
PlGF MoM	–	−0.15	–	−0.713
PAPP-A MoM	–		0.033	0.098
AUC	0.75 (0.64–0.86)	0.76 (0.64–0.87)	0.78 (0.70–0.87)	0.82 (0.74–0.89)

**Table 5:** Regression coefficients and AUC of prediction models for primigravid women (*n* = 71)

Variable	Model with only maternal characteristics	Model with addition of PlGF MoM	Model with addition of PAPP-A MoM	Model with addition of PlGF MoM and PAPP-A MoM
Intercept	17.64	21.96	19.41	14.92
History of hypertension in parents	−1.47	−1.63	−1.49	−1.92
Previous history of hypertension in pregnancy	–	–	–	–
Weight	0.123	0.154	0.134	0.148
Height	−0.216	−0.264	−0.237	−0.214
Parity	–	–	–	–
Diastolic BP	0.110	0.118	0.116	0.118
PlGF MoM	–	0.323	–	0.834
PAPP-A MoM	–	–	0.098	−0.373
AUC	0.899 (0.732–1.000)	0.925 (0.808–1.000)	0.903 (0.749–1.000)	0.951 (0.870–1.000)

## Discussion

The addition of PlGF and PAPP-A together to the model markedly improved its predictive ability, with an increase in AUC from 0.75 to 0.82 for multiparous women and 0.89 to 0.95 for primigravid women, whereas adding either one of the two had only marginal effect. These findings are in line with other studies that reported improved prediction by the addition of biomarkers to maternal characteristics [5, 19, 32–34].

Several issues arise in comparing this study to other prediction studies. The first is that most prediction models predict preeclampsia rather than gestational hypertension [35]. Hence there were fewer prediction models for gestational hypertension to which we could directly compare our models. Therefore we included models for preeclampsia as well in the comparison of the model performance.

The second issue is that we derived separate models for multiparous and primigravid women. This was because the primigravid women could not respond to the question of ”a previous history of gestational hypertension or preeclampsia”. Being an important predictor, we maintained that variable in the model and in a sub analysis fitted a different model for primigravid women (*n* = 71). However because of the relatively small number of primigravid women and outcome events on which these estimates are based, they should be interpreted with caution The third issue is that the same types of biomarkers are not used across prediction studies. Hence finding studies with the same predictors as in this study was a challenge. A number of prediction studies also added uterine artery pulsatility index to biomarkers and maternal characteristics [19, 21, 32] because it improves prediction. For instance, Kuc et al. reported that the best detection rates for preeclampsia were obtained when maternal characteristics, biomarkers and uterine artery pulsatility index were combined [32]. Akolekar et al. also reported a three-fold increase in detection rates in screening for preeclampsia by the combination of maternal factors, biophysical and biomarkers compared with using only maternal factors [19].

Poon et al also reported that PAPP-A and PlGF in combination with maternal characteristics and uterine artery pulsatility index improved detection rates of preeclampsia [21]. We did not include uterine artery pulsatility index in our study because uterine artery Doppler is not readily available in low resource settings.

Another issue is that most of the prediction studies have been conducted in Europe and North America. There are few studies in Sub Saharan African populations to which we could directly compare our results. Ukah et al in a prospective cohort study of pregnant women attending antenatal care in Maputo, Mozambique, measured the serum PlGF concentration in women suspected of having preeclampsia after 20 weeks of gestation. This study had as its primary outcome, the time-to-delivery after confirmation of preeclampsia [36]. This study differed from ours in terms of being a diagnostic study rather than a prediction study.

The AUC is used to quantify the overall ability of a test or a logistic regression model to discriminate between two outcomes such as disease or non-disease [37–40]. It generally ranges from 0.5 to 1 and represents the prediction model’s ability to correctly classify a randomly selected individual as being from one of two hypothetical populations [40–43]. An AUC value of 1.0 is considered perfect, 0.9–0.99 excellent, 0.8–0.89 good, 0.7–0.79 fair and 0.51–0.69 poor. An AUC of 0.5 is considered non-informative. Hence the AUC of 0.82 obtained in our study shows that the model with maternal characteristics and both PAPP-A and PlGF has good predictive ability.

Pencina et al. [44] and Peters et al. [33] have also indicated that increase in the AUC upon the addition of a predictor to a model shows that the predictor has improved the predictive ability of the model. In our study, for the multiparous women, the AUC of the prediction model with only maternal clinical characteristics was 0.75 and this increased to 0.82 upon the addition of both PlGF and PAPP-A to the prediction model. For the primigravid women, the AUC of the prediction model with only maternal clinical characteristics was 0.89 and this increased to 0.95 upon the addition of both PlGF and PAPP-A to the prediction model This is an indication that the addition of both biomarkers simultaneously to the models improved the prediction performance.

The higher median MoM values of PlGF (1.28) and PAPP-A (2.29) in our study compared to the reference population of Dutch women (median MoM of 1 by default) is consistent with other studies that have shown racial and ethnic differences in the levels of these biomarkers, particularly in women of African and Asian decent [45–54]. The median MoM of PAPP-A between 8 weeks gestation to 13 weeks gestation ranged between 1.68 and 4.36. That of PlGF MoM ranged from 0.90 at gestational week 9 to 1.68 at gestational week 13. Differences in the median MoM PlGF and PAPP-A levels between some ethnic groups in Ghana have also been reported in this population [28]. As a result of the higher MoM values, there is the need for a correction factor for the Ghanaian population and sub populations to prevent the under estimation of risk calculations for placental disorders and aneuploidies.

### Clinical and research implications

Hypertensive disorders of pregnancy, including gestational hypertension and preeclampsia, are among the leading causes of maternal morbidity in LMICs. In Ghana they rank as the third leading cause of mortality, having overtaken hemorrhage [26]. The ability to predict this in women at increased risk (of the disorder) and thereby institute preventive measures to minimize their impact is a useful strategy to improving maternal and perinatal outcomes.

Biomarkers have shown some promise in improving the prediction of gestational hypertension and other hypertensive disorders in pregnancy, although a lot more research is still needed. Future studies using larger sample sizes should be conducted to confirm the findings of this study. When confirmed, one factor to be considered in the use of biomarkers in prediction models in the clinical setting would be the cost of carrying out biomarker tests, especially in LMIC settings. A feasible approach in this regard would be the use of dried blood spot samples (DBS) instead of serum which requires refrigeration during storage and transport. DBS have been widely used in newborn screening for sickle cell disease [55, 56], human immunodeficiency virus screening in newborns and for other disorders [57–66]. It is cheaper than conventional serum assay and logistically simpler to implement in screening programmes because samples can be obtained and transported from remote locations where the laboratory infrastructure is limited. The technique for sample taking is also simpler and requires less training compared to venepuncture. In using DBS however, an issue to be considered is how well the concentration of the biomarkers in whole blood correlates with that of DBS. Pennings et al. [67] and Browne et al. [68] have shown that the correlation coefficient between serum and DBS concentrations for PAPP-Aand ß-hCG were both greater than 0.94. Cowans et al also reported that ß-hCG stability is improved in DBS as compared to serum storage. This makes the collection, storage, transport and assay of biomarkers using DBS feasible in low resource settings.

It is recommended that this study should be replicated locally and externally in similar settings using larger sample sizes to validate the findings of this study before possible translation to clinical practice.

The feasibility and sustainability of any planned introduction and eventual scale-up in the use of biomarkers to improve prediction of hypertensive disorders has to be assessed using a cost-benefit analysis.

## Conclusion

The addition of PAPP-A and PlGF to prediction models based on maternal clinical characteristics (diastolic blood pressure, family history of hypertension in parents, history of gestational hypertension in a previous pregnancy, parity, height and weight) markedly improved prediction of gestational hypertension. This study should be replicated using a larger sample size.

## Abbreviations

AUC: Area under the receiver operating characteristic curve
BP: Blood Pressure
DBS: Dried Blood Spot Sample
GH: Gestational Hypertension
HDP: Hypertensive Disorder of Pregnancy
IQR: Inter Quartile Range
LMIC: Low and Middle Income Country
MoM: Multiple of the Median
PAPP-A: Pregnancy Associated Plasma Protein-A
PE: Preeclampsia
PlGF: Placental Growth Factor
RIVM: Dutch Institute for Public Health and Environment
ROC: Receiver Operating Characteristic Curve
SD: Standard Deviation
